# Echinochrome A Treatment Alleviates Fibrosis and Inflammation in Bleomycin-Induced Scleroderma

**DOI:** 10.3390/md19050237

**Published:** 2021-04-23

**Authors:** Gyu-Tae Park, Jung-Won Yoon, Sang-Bin Yoo, Young-Chul Song, Parkyong Song, Hyoung-Kyu Kim, Jin Han, Sung-Jin Bae, Ki-Tae Ha, Natalia P. Mishchenko, Sergey A. Fedoreyev, Valentin A. Stonik, Moon-Bum Kim, Jae-Ho Kim

**Affiliations:** 1 Department of Physiology, Pusan National University School of Medicine, Yangsan 50612, Korea; daramzuy2@naver.com (G.-T.P.); wjddnjssky@nate.com (J.-W.Y.); brown2016@naver.com (S.-B.Y.); dudcjf1114@naver.com (Y.-C.S.); 2 Department of Convergence Medicine, Pusan National University School of Medicine, Yangsan 50612, Korea; parkyong.song@pusan.ac.kr; 3 National Research Laboratory for Mitochondrial Signaling, Department of Physiology, College of Medicine, Cardiovascular and Metabolic Disease Center (CMDC), Inje University, Busan 47392, Korea; estrus74@gmail.com (H.-K.K.); phyhanj@inje.ac.kr (J.H.); 4 Healthy Aging Korean Medical Research Center, Pusan National University, Yangsan 50612, Korea; dr.nowornever@outlook.com; 5 Department of Korean Medical Science, School of Korean Medicine, Pusan National University, Yangsan 50612, Korea; hagis@pusan.ac.kr; 6 G.B. Elyakov Pacific Institute of Bioorganic Chemistry, Far-Eastern Branch of the Russian Academy of Science, 690022 Vladivostok, Russia; mischenkonp@mail.ru (N.P.M.); fedoreev-s@mail.ru (S.A.F.); stonik@piboc.dvo.ru (V.A.S.); 7 Department of Dermatology, Pusan National University School of Medicine, Yangsan 50612, Korea; drkmp@hanmail.net

**Keywords:** echinochrome A, scleroderma, inflammation, fibrosis, systemic sclerosis

## Abstract

Scleroderma is an autoimmune disease caused by the abnormal regulation of extracellular matrix synthesis and is activated by non-regulated inflammatory cells and cytokines. Echinochrome A (EchA), a natural pigment isolated from sea urchins, has been demonstrated to have antioxidant activities and beneficial effects in various disease models. The present study demonstrates for the first time that EchA treatment alleviates bleomycin-induced scleroderma by normalizing dermal thickness and suppressing collagen deposition in vivo. EchA treatment reduces the number of activated myofibroblasts expressing α-SMA, vimentin, and phosphorylated Smad3 in bleomycin-induced scleroderma. In addition, it decreased the number of macrophages, including M1 and M2 types in the affected skin, suggesting the induction of an anti-inflammatory effect. Furthermore, EchA treatment markedly attenuated serum levels of inflammatory cytokines, such as tumor necrosis factor-α and interferon-γ, in a murine scleroderma model. Taken together, these results suggest that EchA is highly useful for the treatment of scleroderma, exerting anti-fibrosis and anti-inflammatory effects.

## 1. Introduction

Scleroderma or systemic sclerosis is an autoimmune disease characterized by deposition of collagen in the connective tissue, resulting in extensive fibrosis in multiple organs and subsequent microvascular dysfunctions [1]. Scleroderma is also associated with the differentiation of fibroblasts to myofibroblasts and an increase in extracellular matrix protein production, such as collagen [2]. Differentiation of fibroblasts into myofibroblasts leads to increased production of extracellular matrix components such as collagen, and the subsequent development of fibrosis. Activated fibroblasts or myofibroblasts, characterized by an increased expression of α-smooth muscle actin (α-SMA) and vimentin, are the key effector cells in scleroderma [1,3]. Fibroblast recruitment and differentiation are regulated by a combination of autocrine and paracrine profibrotic mediators. Despite significant advancements in the treatment of scleroderma, no effective therapy is currently available.

Scleroderma fibrosis is regulated by inflammatory cytokines that are secreted by activated macrophages [4]. Macrophage activation is regulated by inflammatory cytokines and leads to its differentiation between two subtypes, M1 and M2 macrophages [3]. M1 macrophages produce pro-inflammatory mediators, such as TNF-α, IL-1β, and IL-12 [3,4]. Conversely, M2 macrophages produce anti-inflammatory cytokines such as TGF-β [4,5], which stimulates the differentiation of fibroblasts to myofibroblasts. Accumulating evidence suggests that scleroderma is initiated by chronic inflammation, and that increased infiltration and sustained activation of immune cells are responsible for the extensive fibrosis in scleroderma [3]. Moreover, it has been reported that scleroderma can be alleviated by resolution of inflammatory responses [4].

Echinochrome A (6-ethyl-2,3,5,7,8-pentahydroxy-1,4-naphthoquinone, EchA) is a dark-red pigment isolated from sea urchin shells and spines [6] known to have beneficial effects against various diseases, such as middle cerebral artery occlusion, cardiac ischemia, and sepsis [7,8,9]. Through its antioxidant capacity, EchA is known to decrease LPS-induced reactive oxygen species (ROS) production in kidney epithelial cells [10], protect mitochondrial function against cardiac toxicity drugs [11], and ameliorate endotoxin-induced uveitis [12]. EchA treatment also exhibited hepatoprotective effects in septic mice by downregulating oxidative stress in the liver [8] and reduced the severity of bleomycin-induced oxidative stress and fibrosis in the lungs [13]. These reports suggest that EchA has therapeutic effects against many types of oxidative stress-related diseases. Recently, it has been reported that EchA exerts therapeutic effects in dextran sodium sulfate-induced colitis through the downregulation of T-cell polarization, thus augmenting regulatory T cell generation, and increasing macrophage M2 polarization [14]. Although EchA has an immunomodulatory effect, it is still unclear whether it has therapeutic effects on scleroderma. In this study, we explored the therapeutic and anti-fibrotic effects, as well as the inflammatory regulation effect, of EchA in a bleomycin-induced scleroderma animal model.

## 2. Results

### 2.1. EchA Treatment Alleviates Fibrosis in Bleomycin-Induced Scleroderma

**Figure 1:**
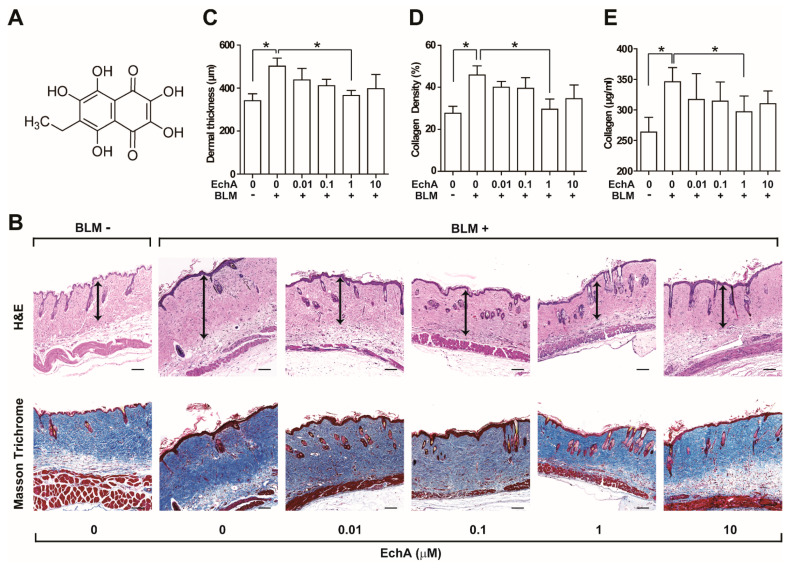
Dose-dependent effects of EchA treatment on tissue fibrosis in a scleroderma mice model. (**A**) Chemical structure of EchA. (**B**) Dose-dependent effect of Echinochrome A (EchA) on BLM-induced scleroderma. The BLM-induced scleroderma mice (BLM+) were treated with the increasing dose of EchA daily for an additional three weeks, and skin sections were stained using H&E and Masson’s trichrome staining kits. The dermal layer between the epidermal–dermal junction and the dermal–fat junction is indicated by an arrow on H&E-stained sections. Dermal thickness (**C**) and collagen density (**D**) were quantified from the H&E and Masson’s trichrome data, respectively. (**E**) Effect of EchA on the BLM-induced increase in hydroxyproline content in skin. Data represent the mean ± SD (*n* = 5 per group). * *p* < 0.05.

To establish a scleroderma animal model, mice were injected subcutaneously with bleomycin daily for 3 weeks. Bleomycin treatment resulted in an increase in dermal thickness, collagen density, and hydroxyproline levels, which are indicators of fibrosis [15]. To determine the effect of EchA on tissue fibrosis, increasing doses of EchA were subcutaneously co-administered with bleomycin for 3 weeks (Figure 1). The chemical structure of EchA is shown in Figure 1A. EchA treatment dose-dependently reduced the bleomycin-induced increase in dermal thickness with maximal reduction at 1 μM (Figure 1B,C). The BLM-induced scleroderma skin exhibited excessive deposition of collagen fibers as demonstrated by Masson’s trichrome staining (Figure 1B). Consequently, the BLM-induced increase in collagen density was significantly alleviated by co-administration with EchA (Figure 1D). Moreover, the levels of hydroxyproline, which are indicators of collagen deposition, were upregulated by BLM treatment, and co-administration with EchA markedly reduced these levels (Figure 1E). However, administration of EchA alone did not affect the collagen content in normal skin tissue (Figure S1).

**Figure 2:**
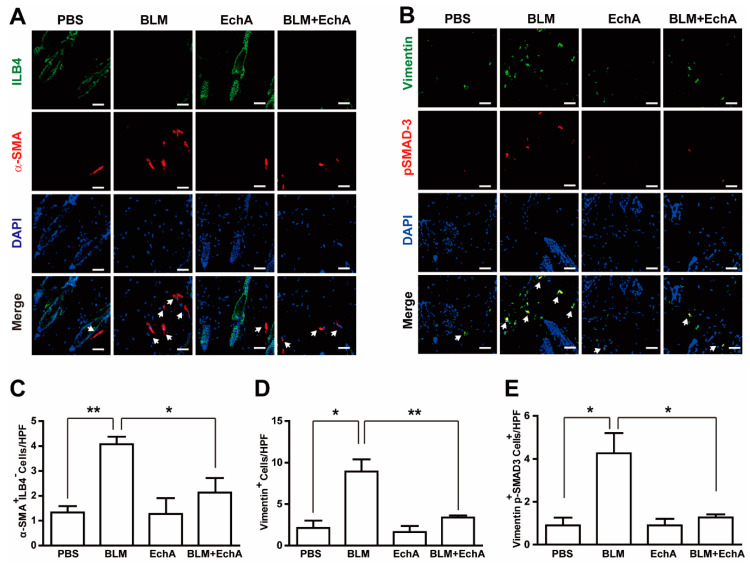
Effects of EchA treatment on myofibroblast differentiation in scleroderma. (**A**) EchA-induced inhibition of myofibroblast differentiation in scleroderma. The BLM-induced scleroderma mice were treated with or without 1-µM EchA for three weeks, and the mock-treated (PBS) and the scleroderma (BLM or BLM+EchA) skin specimens were immunostained with antibodies against α-SMA and ILB-4. (**B**) The skin specimens were stained with antibodies against vimentin and phospho-SMAD3. Scale bar = 50 μm. The numbers of α-SMA^+^ILB-4^−^ (**C**), vimentin^+^ (**D**), and vimentin^+^phospho-SMAD3^+^ (**E**) cells were quantified under a high-power field. Data represent the mean ± SD (*n* = 4 per group). * *p* < 0.05, ** *p* < 0.01 .

It has been reported that fibroblast activation and differentiation toward myofibroblasts is a crucial process for developing scleroderma [1,3]. Although α-SMA is a myofibroblast marker, it is also expressed in blood vessels. To discriminate myofibroblasts from the blood vessels, the skin specimens were co-stained with antibodies against α-SMA as well as ILB-4, which is a marker of endothelial vascular cells, and the numbers of α-SMA^+^ILB4^-^ myofibroblasts were counted. The number of α-SMA^+^ILB4^-^ myofibroblasts increased in BLM-treated mice, and EchA co-administration prevented this (Figure 2A,C). Moreover, the number of vimentins, a marker of fibroblast activation, was also increased in BLM-treated mice skin, and EchA treatment reduced the number of vimentin^+^ cells as well (Figure 2B,D). In fibrotic disease, TGF-β–dependent SMAD3 phosphorylation has been reported to play a key role in fibroblast activation and differentiation [16]. To further confirm whether EchA regulates fibroblast differentiation, we co-stained the skin specimens with antibodies against phospho-SMAD3 and vimentin. Consistently, the number of Vimentin^+^p-SMAD3^+^ cells was significantly increased in BLM-treated mice, and EchA treatment completely normalized the number of Vimentin^+^pSMAD3^+^ cells in BLM-treated mice skin (Figure 2B,E). Consistently, EchA abrogated the BLM-induced expression of ACTA2 (α-SMA), CCN2, COL1A1, and COL1A2, which are implicated in fibrosis (Figure S2). Moreover, EchA treatment abrogated the TGF-β1-induced expression of α-SMA and p-Smad in dermal fibroblasts in vitro (Figure S3). Taken together, these results suggested that EchA has anti-fibrotic effects in a BLM-induced scleroderma mouse model.

### 2.2. EchA Treatment Attenuates Inflammation in BLM-Induced Scleroderma

**Figure 3:**
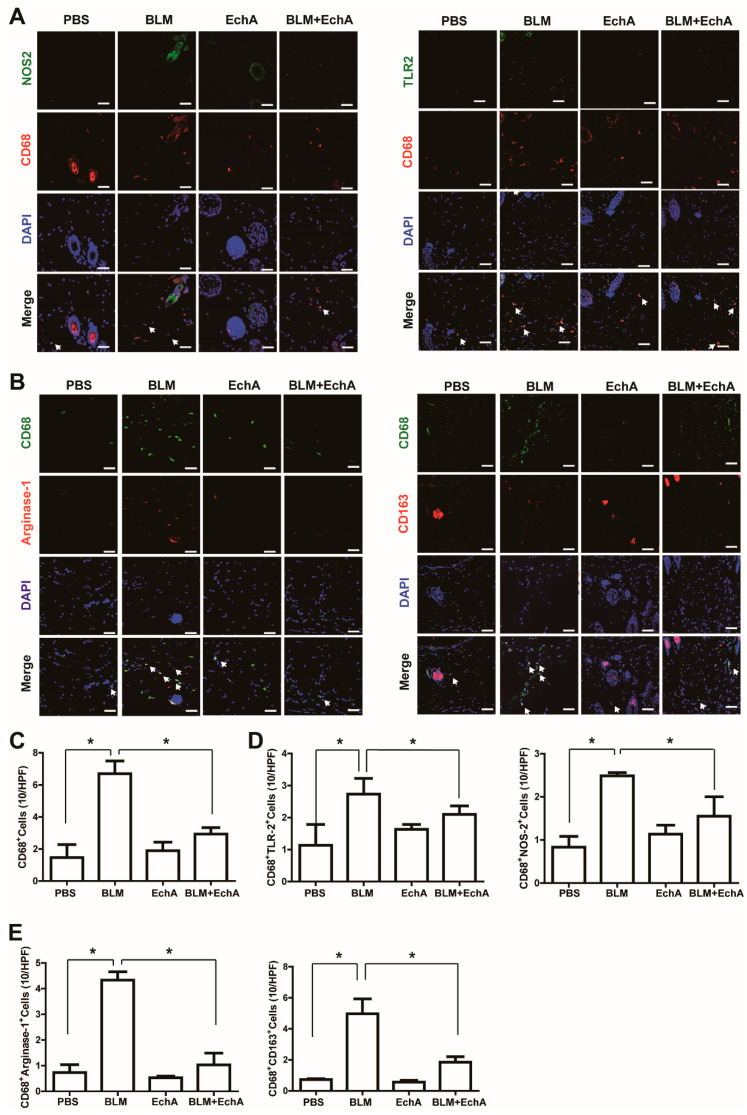
Effects of EchA treatment on the number of macrophages and inflammation in scleroderma. The BLM-induced scleroderma mice were treated with or without EchA for three weeks. The mock-treated (PBS) and scleroderma (BLM or BLM+EchA) skin specimens were stained with anti-CD68 antibody together with anti-NOS2 or anti-TLR2 (**A**), anti-Arginase-I or CD163 (**B**) antibodies. Nuclei were stained with DAPI and overlaid images are shown. Scale bar = 50 μm. The numbers of CD68^+^ macrophages (**C**), M1 type macrophages ((**D**); CD68^+^TLR2^+^ and CD68^+^NOS2^+^ cells), M1 type macrophages ((**E)**; CD68^+^Arginse-1^+^ and CD68^+^CD163^+^ cells) were counted under high-power field. Data represent the mean ± SD (*n* = 4 per group). * *p* < 0.05.

Inflammatory cell activation and infiltration into skin tissue has been reported to induce systemic sclerosis under chronic inflammation. To explore the effects of EchA treatment on inflammatory action associated with scleroderma, we measured the levels of macrophage infiltration and activation using immunohistochemistry analysis. The number of CD68-positive macrophages increased in the BLM-induced scleroderma skin compared with the control, and EchA co-treatment suppressed BLM-induced macrophage infiltration (Figure 3A,C). Activation of macrophages is known to induce polarization toward pro-inflammatory M1 macrophages or anti-inflammatory M2 macrophages [3]. Thus, we measured the numbers of M1 and M2 macrophages in the skin specimens by immunostaining. The number of M1 macrophages, which include CD68^+^TLR2^+^ and CD68^+^NOS2^+^ cells, were increased in BLM-treated scleroderma skin, and EchA co-treatment reduced the number of M1 macrophages (Figure 3A,D). Moreover, the numbers of M2 macrophages, namely CD68^+^Arginase-I^+^ and CD68^+^CD163^+^ macrophages, were increased by BLM treatment and EchA co-treatment abrogated this increase (Figure 3B,E). These results suggest that EchA attenuates inflammatory cell activation and infiltration into BLM-induced scleroderma tissues.

### 2.3. EchA Treatment Attenuates Systemic Inflammation in BLM-Induced Scleroderma

**Figure 4:**
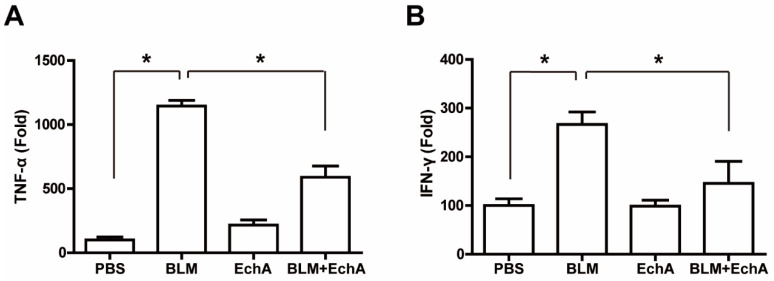
The role of EchA-induced immune cytokine resolution in scleroderma. (**A**,**B**) EchA-induced inhibition of systemic inflammation in scleroderma. The BLM-induced scleroderma mice were treated with or without EchA for three weeks, and the serum levels of TNF-α (**A**) and INF-γ (**B**) in the mock-treated (PBS) and the scleroderma mice (BLM or BLM+EchA) were determined by ELISA assay. Data represent the mean ± SD (*n* =4 per group). * *p* < 0.05.

In scleroderma, inflammatory cytokines are increased in peripheral blood, which in turn activate inflammatory cells. To explore whether EchA treatment affects inflammatory cytokine levels in scleroderma mice, serum levels of TNF-α and IFN-γ were measured by ELISA in a BLM-induced scleroderma model. The serum levels of TNF-α and INF-γ in BLM-treated mice were greater than those in PBS-treated control mice. EchA co-treatment markedly attenuated the BLM-induced increase in TNF-α and IFN-γ levels (Figure 4), suggesting that EchA administration reduces systemic inflammation in BLM-induced scleroderma.

## 3. Discussion

Systemic sclerosis is a chronic autoimmune disease that induces multiple organ fibrosis [1]. It activates inflammatory cells and promotes cytokine production, which consequently induces the differentiation of fibroblasts to myofibroblasts and promotes extensive production of extracellular matrix [2]. BLM exerts anti-tumorigenic effects by inducing DNA strand destruction in cancer cells [17,18]; however, it has deadly side effects, causing fibrosis in patients [19,20]. BLM has been shown to increase ROS production in pulmonary alveolar tissues and induce inflammatory cell activation, such as macrophage activation and infiltration in connective tissues [21]. Moreover, BLM-induced ROS is implicated in the conversion of TGF-β from a latent to an active form in alveolar epithelial cells [22]. Consequently, TGF-β induces SMAD2/3 phosphorylation and promotes the expression of fibrotic genes, such as Col1a1, Col3a1, TIMP1, and α-SMA [23]. In this report, we demonstrated the anti-fibrotic effects of EchA in BLM-induced scleroderma mice. EchA has been reported to reduce the severity of BLM-induced oxidative stress and fibrosis in lungs [13]. Moreover, it has been reported that TGF-β and CTGF levels are upregulated by tissue factor-thrombin-dependent coagulation [24,25], which is enhanced in SSc patients [26,27]. We found that the tissue factor level was upregulated in sera of BLM-induced scleroderma mice, and that EchA treatment reduced the BLM-induced increase in tissue factor (Figure S4). These results suggest that EchA administration prevents scleroderma-associated fibrosis by attenuating TGF-β production which is dependent on ROS and tissue factor.

Accumulating evidence suggests that antioxidant drugs have therapeutic effects on scleroderma. For instance, N-acetylcysteine, a well-known antioxidant compound, has therapeutic effects in scleroderma through antioxidant enzyme activation and oxidative stress inhibition [28]. In addition, the antioxidant flavonoid Kaempferol inhibits fibroblast activation and inflammatory cell infiltration in a bleomycin-induced scleroderma mouse model [29]. EchA possesses potent antioxidant, antimicrobial, anti-inflammatory, and chelating abilities [10,30], and has been reported to ameliorate LPS-induced uveitis by reducing ROS production, NF-κB expression, and TNF-α expression [12]. In patients with cardiovascular diseases, clinical trials with EchA administration effectively recovered antioxidant status and reduced atherosclerotic inflammation [26]. Moreover, EchA administration protected the liver from sepsis-induced liver damage by counteracting hepatic oxidative stress [8]. EchA is known to protect mitochondrial function in cardiomyocytes against cardiotoxic drugs by attenuating oxidative stress [11,26]. Therefore, these results suggest that the antioxidant activities of EchA may be implicated in the EchA-mediated therapy of scleroderma.

Macrophage-derived TGF-β has been known to stimulate myofibroblasts to produce various extracellular matrix proteins and inhibitors of metalloproteases that are associated with tissue fibrosis [31,32,33]. Notably, M1 macrophages are involved in inflammation and tissue damage responses, while M2 macrophages are primarily linked with the pathogenesis and fibrosis associated with scleroderma [34]. In the present study, we demonstrated that EchA administration attenuated the number of macrophages infiltrated into scleroderma tissues, and the numbers of not only M1 but also M2 type macrophages were reduced by EchA treatment. We have previously reported that activation of formyl peptide receptor 2 ameliorated dermal fibrosis by reducing the number of M2 macrophages in BLM-induced scleroderma [35]. These results suggest that EchA-induced suppression of inflammatory responses, including macrophage activation, plays a key role in the therapy of scleroderma. It has been shown that neutrophil extracellular traps, which are extracellular DNA fibers implicated in immune protection and autoimmune diseases, are increased in systemic sclerosis patients [31]. Neutrophil extracellular traps are also involved in fibroblast activation [32]. We found that EchA treatment reduced the BLM-stimulated neutrophil extracellular trap levels in vivo (Figure S5). Moreover, it has been reported that autophagy is key feature in the pathogenesis of systemic sclerosis [36], and that autophagy of macrophages is implicated in the TGF-β-induced fibrosis [18,37]. In the present study, we found that the LC3b level is downregulated in the BLM-treated macrophages, suggesting BLM-induced autophagy; however, EchA treatment did not significantly affect the BLM-induced autophagy of macrophages (Figure S6). Therefore, it is likely that therapeutic effects of EchA are achieved by regulating neutrophil extracellular traps, not autophagy, in inflammatory cells. In conclusion, the present study demonstrates that EchA treatment alleviates fibrosis by inhibiting fibroblast activation, inflammation, and inflammatory cytokine expression in scleroderma mouse models. These results suggest EchA can be useful for the treatment of patients with scleroderma and other autoimmune diseases.

## 4. Materials and Methods

### 4.1. Materials

BLM was purchased from Tokyo Chemical Industry (Tokyo, Japan). Anti-α-SMA (ab5694) and anti-CD163 antibodies (ab182422) were purchased from Abcam PLC (Cambridge, UK). Anti-CD68 antibody (MCA1957GA) was purchased from AbD Serotec (Raleigh, NC, USA). Anti-NOS2 antibody (SC-650) was purchased from Santa Cruz Biotechnology, Inc. (Dallas, TX, USA). Anti-phospho-SMAD3 antibody (44-246G), anti-Arginase-I antibody (PA5-29645), and anti-TLR2 antibody (PA5-20020) were purchased from Thermo Fisher Scientific (Waltham, MA, USA). Anti-vimentin antibody (MAB-2105SP) was purchased from R&D Systems (Minneapolis, MN, USA). Mouse enzyme-linked immunosorbent assay (ELISA) kits for TNF-α (430904) and INF-γ (430804) were purchased from BioLegend (San Diego, CA, USA). The QuickZyme Total Collagen Assay kit was purchased from QuickZyme Biosciences (Leiden, The Netherlands).

### 4.2. Isolation of EchA

Ech A was isolated from the sea urchins *Scaphechinus mirabilis*, which were collected in Peter the Great Bay in the Sea of Japan, Russia. The sea urchins were extracted with EtOH containing H_2_SO_4_ (10%). The extract was concentrated in vacuo and distributed between H_2_O and CH_3_Cl. The CH_3_Cl fraction was evaporated and chromatographed over columns of a Toyopearl HW-40 (TOYOSODA, Tokyo, Japan) using EtOH–H_2_O solvents containing formic acid (0.01%) with a gradient of EtOH increasing from 10 to 50%. Then, EchA was crystallized from EtOH. The purity of EchA was measured as 99.0% by HPLC analysis. Detailed information about NMR spectra and HPLC analysis of EchA was described in Supplementary Information.

### 4.3. Scleroderma Animal Model

C57BL6/J mice (six weeks old, weighing 22–24 g, male) were purchased from Koatech (Gyeonggi-do, Korea). To establish the scleroderma animal model, the backs of the C57BL6/J mice were shaved and subcutaneously injected with 100 μL BLM solution (1 mg/mL) using a 27-gauge needle at a single location (1 cm^2^) for three weeks. To explore the therapeutic effects of EchA on scleroderma, EchA was administered into skin together with BLM for three weeks. The therapeutic effects of EchA on scleroderma were examined by histological analysis of the scleroderma skin tissues of mice. All animals were housed in an air-conditioned animal room with constant relative humidity and were provided a standard laboratory diet and water. Animal treatment and maintenance were performed in accordance with the Principles of Laboratory Animal Care, and animal experiments were performed using protocols approved by the Pusan National University Institutional Animal Use and Care Committee (PNU-2019-2400).

### 4.4. Histological Analysis

For histological analysis of scleroderma tissues, the animals were sacrificed, and skin specimens were excised. The skin samples were fixed in 4% paraformaldehyde overnight and embedded in paraffin. The specimens were sectioned and stained with hematoxylin and eosin (H&E) for determination of dermal thickness, and five areas in one section were randomly selected for quantitative analysis of histological data. Images of stained tissue sections were scanned using Axio Scan.Z1 (Carl Zeiss Microscopy, Germany) at 100× magnification. Using Image J, the dermal thickness or distance between the epidermal–dermal and the dermal–subcutaneous fat junctions were measured on H&E-stained images. To determine the amount of collagen deposition, tissue sections were stained with Masson’s trichrome staining, and collagen density in skin specimens was quantified using Image J analysis of the stained sections. Collagen deposition in scleroderma skin tissues was measured using a hydroxyproline assay kit (QuickZyme Total Collagen Assay kit) according to the manufacturer’s protocol.

### 4.5. Immunohistological Analysis

Formalin-fixed, paraffin-embedded skin sections were stained with antibodies against α-SMA, vimentin, and phospho-SMAD3 for the visualization of myofibroblasts. Anti-ILB4 antibody and anti-CD68 antibody were used for staining of endothelial cells and macrophages, respectively. Anti-NOS2 and anti-TLR2 antibodies were used for staining of M1 macrophages, and M2 macrophages were stained with anti-CD163 and anti-Arginase-I antibodies. The tissue specimens were then incubated with Alexa Fluor 488 goat anti-rabbit, Alexa Fluor 568 goat anti-rabbit, or Alexa Fluor 488 goat anti-rat antibody, and then washed and mounted in Vectashield medium containing 4′,6-diamidino-2-phenylindole (DAPI) for staining of nuclei. The stained sections were then visualized under a laser confocal microscope (Olympus FluoView FV1000). The numbers of α-SMA^+^ILB4^−^, Vimentin^+^, and Vimentin^+^p-SMAD3^+^ myofibroblasts, and the numbers of CD68^+^ macrophages, both M1 type (CD68^+^NOS2^+^ and CD68^+^TLR2^+^) and M2 type (CD68^+^Arginase-I^+^ and CD68^+^CD163^+^), were quantified in a high-power field using ImageJ software. Four randomly selected microscopic fields from three serial sections in each tissue block were examined by two independent observers blinded to the experimental conditions.

### 4.6. ELISA Assay

For the determination of cytokine levels in mouse sera, blood was collected from mice by cardiac puncture and centrifuged at 1000× *g* for 30 min at 4 °C. TNF-α and IFN-gamma levels were measured using ELISA kits (BioLegend). Tissue factor levels in serum were measured using a mouse Tissue Factor ELISA kit (Abcam, Cambridge, UK). The absorbance values were measured at 450 nm using a microplate reader (TECAN Life Sciences, Männedorf, Switzerland).

### 4.7. Autophagy Assay

For the determination of autophagy activity in macrophages, pMX retroviral vector expressing Luciferase-rLC3b (Luc-rLC3b) was constructed for production of retrovirus. Raw 264.7 mouse macrophages were infected with the retrovirus expressing Luc-rLC3b, and cultured in DMEM supplemented with 1% Penicillin/Streptomycin and 10% FBS in 5% CO_2_ at 37 °C incubator. The Luc-rLC3b-expressing Raw 264.7 cells (1 × 10^5^/well) were seeded in a 24-well plate, serum starved at DMEM basal media for 2 h, followed by treatment with BLM and EchA for 2 h. The autophagy activity in cell lysates was measured by using luciferase reporter assay system (Promega, Madison, WI, USA) and measured by a Victor3 Multilabel Plate Reader (PerkinElmer, Waltham, MA, USA).

### 4.8. Real-Time PCR (RT-PCR)

Total RNA was isolated from mouse dermal tissue using TRIsure (BI0-38033, Bioline Co., Ltd., London, UK). Purity and concentration of RNA were measured by pectrophotometer (Optizen, K Lab Co., Ltd., Korea) and then 1 μg of RNA were synthesized into cDNA using the Reverse Transcription cDNA Kit (#RT50KN; NanoHelix Co., Ltd, Daejeon, Korea.). cDNA (1 μL each) was added into a PCR tube containing HelixAmpTM Ready-2×GO (NanoHelix, Co., Ltd.) and 10 pmol of sense and antisense primer, and RT-PCR was performed using an ABI (Applied Biosystems, Foster City, CA) sequence detection system with SYBR Green Master Mix (ABS-4309155, Applied Biosystems, Foster City, CA) according to the manufacturer’s instructions. Data were analyzed using the Δ(ΔCT) method and normalized to GAPDH. Primer sequences are listed in Supplementary Table S1.

### 4.9. Western Blotting

Human Dermal Fibroblasts were isolated from donors and incubated in alpha MEM with 1% Penicillin/Streptomycin and 10% FBS in 5% CO_2_ at 37 °C incubator. Fibroblasts (2 × 10^5^/well) were seeded in a 6-well plate, serum starved in alpha MEM basal media for 24 h. Cells were lysed in a lysis buffer (pH 7.4; 20 mM Tris-HCl, 1 mM EGTA, 1 mM EDTA, 10 mM NaCl, 0.1 mM phenylmethyl sulfonyl fluoride, 1 mM Na3VO4, 30 mM sodium pyrophosphate, 25 mM β-glycerol phosphate, and 1% Triton X-100). Lysates were resolved by sodium dodecyl sulfate-polyacrylamide gel electrophoresis (SDS-PAGE) and the proteins were transferred to nitrocellulose membranes. The proteins were stained with 0.1% Ponceau S solution (Sigma-Aldrich Co. Ltd., St. Louis, MO, USA), blocked with 5% non-fat milk, and immunoblotted with anti-α-SMA, anti-Smad3, anti-p-Smad3 and anti-GAPDH antibodies overnight. Bound antibodies were visualized with horseradish peroxidase-conjugated secondary antibodies using an Enhanced Chemiluminescence system (GE Healthcare Life Sciences, Pittsburgh, PA, USA).

### 4.10. Statistical Analysis

The results of multiple observations are presented as the mean ± SD. For multivariate data analysis, group differences were assessed using one-way or two-way ANOVA, followed by Scheffé’s post hoc test.

## Data Availability

The data presented in this study are available on request from the corresponding author.

## Supplementary Materials

The following are available online at https://www.mdpi.com/article/10.3390/md19050237/s1, Table S1: Primer lists for RT-PCR analysis, Figure S1: Therapeutic effects of EchA treatment on tissue fibrosis in scleroderma mice model, Figure S2: Effects of EchA treatment on the expression of fibrosis markers in scleroderma mice model, Figure S3: Effects of EchA treatment on TGF-β1-induced myofibroblast differentiation of human dermal fibroblasts, Figure S4. Effects of EchA on the expression of tissue factor in scleroderma, Figure S5. Effects of EchA treatment on neutrophil extracellular traps in scleroderma, Figure S6. Effects of EchA and bleomycin on autophagy of macrophages.
