# Supplementary material for: Echinochrome A Treatment Alleviates Fibrosis and Inflammation in Bleomycin-Induced Scleroderma
Source: Mar Drugs. 2021 Apr 23;19(5):237. doi: 10.3390/md19050237 (PMC8146844; doi:10.3390/md19050237)
Supplement: Supplementary file 1 [file marinedrugs-19-00237-s001.zip › marinedrugs-1181642-supplementary.pdf]

Supporting Information

Echinochrome A treatment alleviates fibrosis and inflammation in bleomycin-induced scleroderma

Gyu Tae Park[1*], Jung Won Yoon[1*], Sang Bin Yoo[1], Young Chul Song[1], Parkyong Song[2], Hyoung Kyu Kim[3], Jin Han[3], Sung-Jin Bae [4], Ki-Tae Ha [5], Natalia P Mishchenko [6], Sergey A Fedoreyev [6], Valentin A Stonik [6], Moon Bum Kim [7], and Jae Ho Kim[1‡]

[1]Department of Physiology, Pusan National University School of Medicine, Yangsan 50612, Republic of Korea.

[2]Department of Convergence Medicine, Pusan National University School of Medicine, Yangsan 50612, Republic of Korea.

[3]National Research Laboratory for Mitochondrial Signaling, Department of Physiology, College of Medicine, Cardiovascular and Metabolic Disease Center (CMDC), Inje University, Busan 47392

[4]Healthy Aging Korean Medical Research Center, Pusan National University, Yangsan 50612, Republic of Korea

[5]Department of Korean Medical Science, School of Korean Medicine, Pusan National University, Yangsan 50612, Republic of Korea

[6]G.B. Elyakov Pacific Institute of Bioorganic Chemistry, Far-Eastern Branch of the Russian Academy of Science, Vladivostok 690022, Russia

[7]Department of Dermatology, Pusan National University School of Medicine, Yangsan 50612, Republic of Korea.

Supplementary Materials and Methods

Echinochrome A properties

Echinochrome A (EchA), $C_{12}H_{10}O_7$, red-brown needles, m.p. 220-221 °C.

UV-Vis spectrum of a 0.002% solution of echinochrome A in EtOH·1mM HCl has λ_{max} at 342 and 468 nm, λ_{min} at 295 and 392 nm, and two shoulders from 485 to 500 nm and from 515 to 537 nm.

NMR spectra were recorded on a Bruker Avance III 700 spectrometer (700 MHz for ^{1}H and 176 MHz for ^{13}C).

^{1}H NMR ($CDCl_3$), δ: 1.17 (t, 3 H, Me, J = 7.5 Hz); 2.73 (q, 2 H, CH_2, J = 7.5 Hz); 6.36 (s, 1 H, C(3)OH); 6.52 (s, 1 H, C(6)OH); 6.80 (s, 1 H, C(2)OH); 12.06 (s, 1 H, C(5) OH); 12.27 (s, 1 H, C(8)OH).

^{13}C NMR ($CDCl_3$), δ: 12.0 (CH_3); 16.2 (CH_2); 101.8 (C(9)); 106.3 (C(10)); 126.0 (C(7)); 135.8 (C(2)); 138.3 (C(3)); 148.7 (C(5)); 151.3 (C(6)); 161.3 (C(8)); 178.00 (C(1)); 180.0 (C(4)).

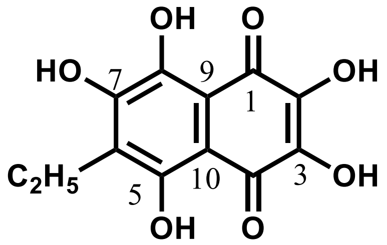

ESI-MS, m/z: 265 $[M-H]^-$, 267 $[M+H]^+$ (Shimadzu LCMS-2020, Shimadzu Corp., Kyoto, Japan).

The purity of Ech A (99.0%) was confirmed by HPLC analysis.

HPLC Analysis. The Agilent Technologies 1100 Series HPLC system (Agilent Technologies Deutschland GmbH, Waldbronn, Germany) were used for analytical HPLC. A ZORBAX Eclipce XDB-C8 column (150 ×4.6 mm, 5 mkm) was used. The mobile phase was 1% aqueous acetic acid (A) and acetonitrile containing 1% acetic acid (B) with a gradient elution of 10-30% B (6 min), 30-70% B (20 min), and 70-30% B (25 min). The flow rate was 1.0 mL/min at 30 °C and monitored by UV at 254 nm. The retention time (tR) value for EchA was 11.1 min.

Table S1. Primer lists for RT-PCR analysis

Gene	Sequence from 5′ to 3′	
GAPDH	Forward	GCA GTG GCA AAG TGG AGA TT
	Reverse	CAC ATT GGG GGT AGG AAC AC
ACTA2	Forward	CCT GAC GGG CAG GTG ATC
	Reverse	ATG AAA GAT GGC TGG AAG AGA GTC T
CCN2	Forward	AAA GTG CAT CCG GAC ACC TAA
	Reverse	TGC AGC CAG AAA GCT CAA ACT
COL1A1	Forward	TTC GGA CTA GAC ATT GG
	Reverse	GGG TTG TTC GTC TGT TTC
COL1A2	Forward	CCG TGC TTC TCA GAA CAT CA
	Reverse	CTT GCC CCA TTC ATT TGT CT

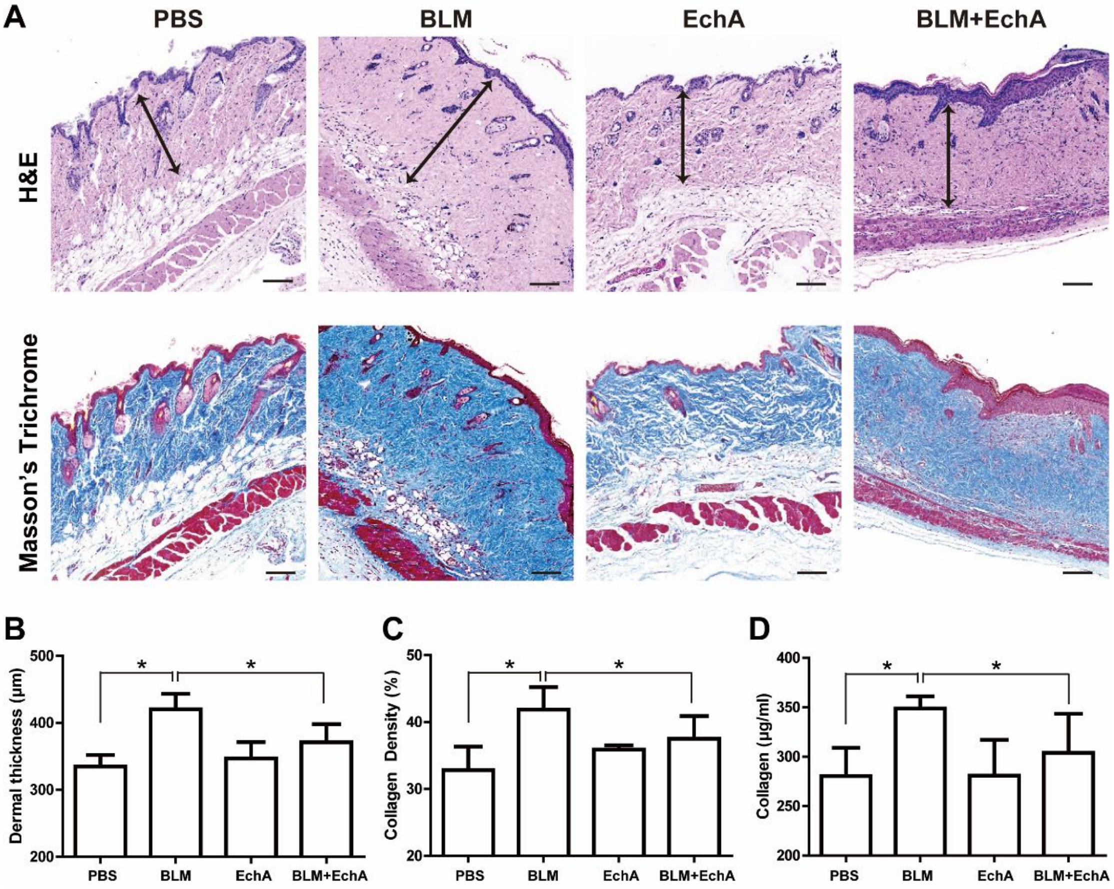

Figure S1. Therapeutic effects of EchA treatment on tissue fibrosis in scleroderma mice model. (A) Therapeutic effects of EchA-induced repair of scleroderma. PBS, BLM, EchA, and BLM+EchA were introduced to the mice for three weeks. The mock-treated (PBS) and the scleroderma (BLM or BLM+EchA) skin specimens were stained using H&E and Masson's trichrome staining kits. The dermal layer between the epidermal–dermal junction and the dermal–fat junction is indicated by an arrow on H&E-stained sections. Dermal thickness (B) and collagen density (C) were quantified from the H&E and Masson's trichrome staining data, respectively. (D) The effects of EchA on the levels of hydroxyproline content in skin specimens were determined. Data are shown as the mean ± SD (n=5 per group). *p<0.05.

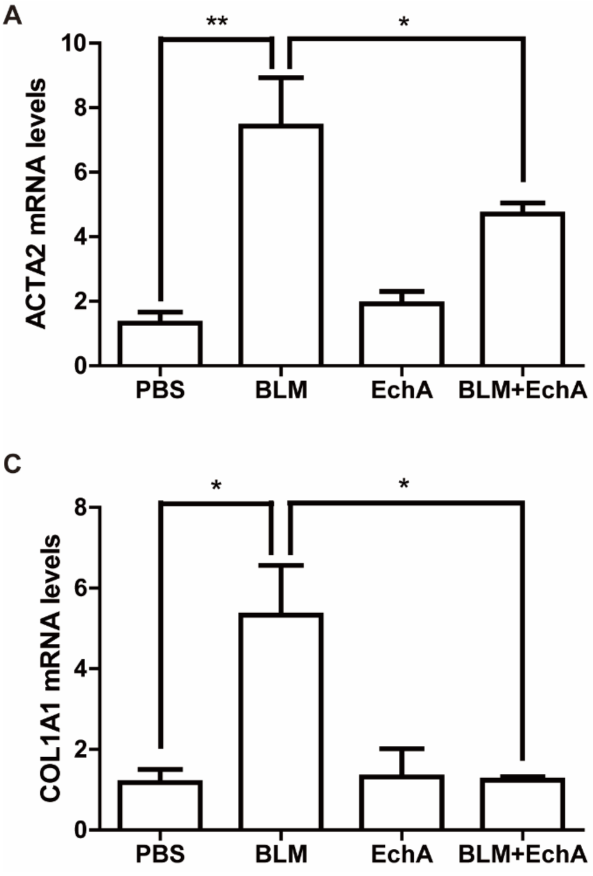

Figure S2. Effects of EchA treatment on the expression of fibrosis markers in scleroderma mice model. The BLM-induced scleroderma mice models were treated with or without EchA (1 μM) for three weeks, and the mRNA levels of ACTA2 (A), CCN2 (B), COL1A1 (C) and COL1A2 (D) in the skin tissues of the mock-treated (PBS) and the scleroderma mice (BLM or BLM+EchA) were determined by real time RT-PCR analysis. Data are shown as the mean ± SD (n = 5 per group). $*p < 0.\ 05$, $**p < 0.01$, $***p < 0.005$.

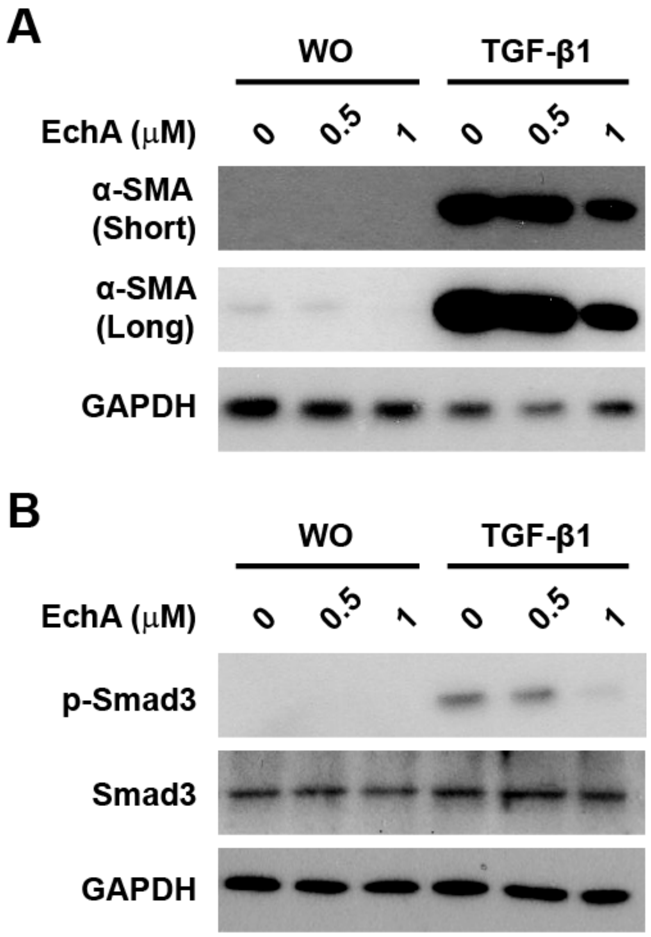

Figure S3. Effects of EchA treatment on TGF-β1-induced myofibroblast differentiation of human dermal fibroblasts. Human dermal fibroblasts were treated with or without TGF-β1(5 ng/ml) in the presence of EchA (0, 0.5, 1 μM) for 4 days (A) and 30 min (B). The protein levels of α-SMA, Smad3, p-Smad3, and GAPDH in Dermal fibroblasts were determined by Western blotting. Shorter or longer exposure of α-SMA protein bands are shown.

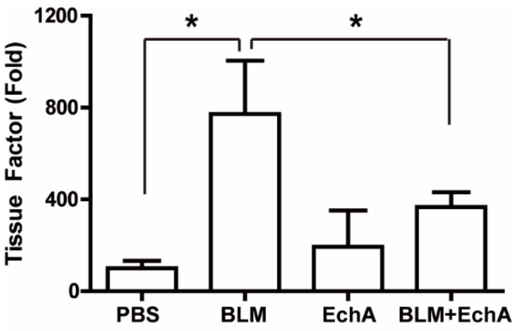

Figure S4. Effects of EchA on the expression of tissue factor in scleroderma. The BLM-induced scleroderma mice were treated with or without EchA (1 μM) for three weeks, and the serum levels of Tissue Factor in the mock-treated (PBS) and the scleroderma mice (BLM or BLM+EchA) were determined by ELISA assay. Data represent mean ± SD (n =4 per group). *$p < 0.05$.

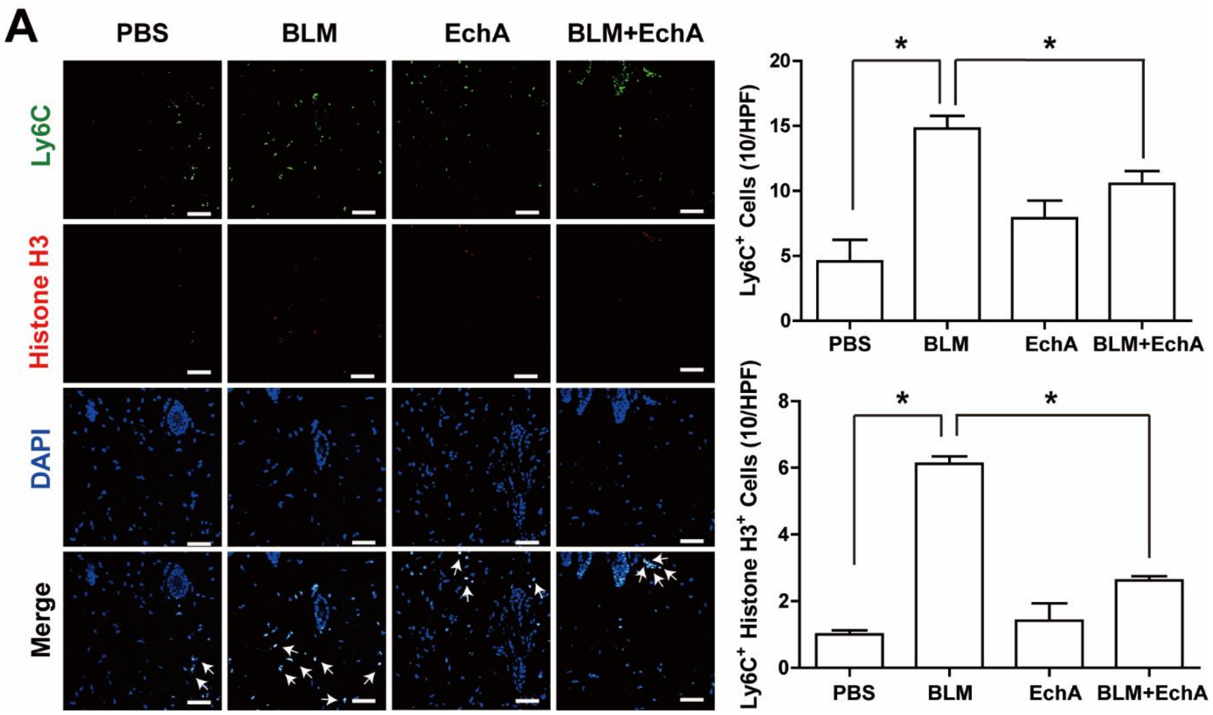

Figure S5. Effects of EchA treatment on neutrophil extracellular traps in scleroderma. The BLM-induced scleroderma mice were treated with or without EchA (1 μM) for three weeks. The mock-treated (PBS) and scleroderma (BLM or BLM+EchA) skin specimens were stained with anti-Ly6C antibody together with anti-Histone H3 (A) antibodies. Nuclei were stained with DAPI and overlaid images are shown. Scale bar = 50 μm. The numbers of Ly6C^{+} neutrophils (B), Neutrophil extracellular traps-forming neutrophils (C; Ly6C^{+}Histone-H3^{+} cells) were counted under high-power field. Data represent mean ± SD (n = 4 per group). *$p <$ 0.05.

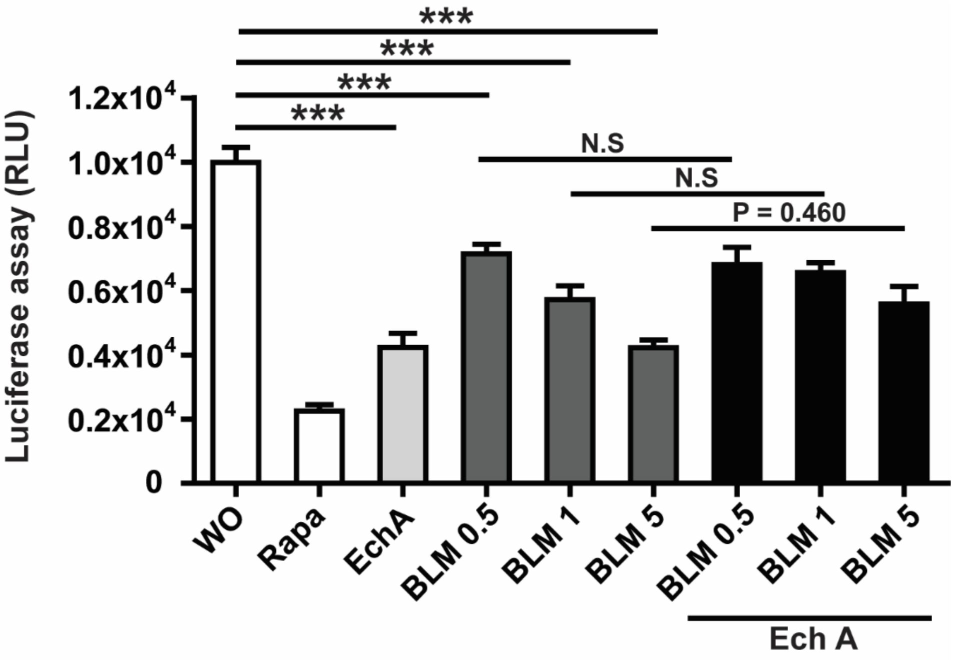

Figure S6. Effects of EchA and bleomycin on autophagy of macrophages. Raw 264.7 macrophages were transfected with Luc-rLc3b plasmid, and treated with rapamycin (200 nM), EchA (1 μM), bleomycin (0.5, 1, 5 μg/ml), and EchA plus bleomycin for 2 h. Reduced luciferase activity indicated degradation of Lc3b and activation of autophagy. Both bleomycin and EchA induced autophagy in macrophages, and BLM-induced autophagy was not affected by EchA co-treatments. Luc-LC3b level was measured by using luciferase reporter assay system and a Victor3 multilabel plate reader. Data represent mean ± SD (n =4 per group). ***p < 0.005. N.S., not significant.
